# Butyrate suppresses demyelination and enhances remyelination

**DOI:** 10.1186/s12974-019-1552-y

**Published:** 2019-08-09

**Authors:** Tong Chen, Daisuke Noto, Yasunobu Hoshino, Miho Mizuno, Sachiko Miyake

**Affiliations:** 1 Department of Immunology, Juntendo University School of Medicine, Bunkyo-ku, Tokyo, Japan; 2 Department of Neurology, Juntendo University School of Medicine, Bunkyo-ku, Tokyo, Japan

**Keywords:** Short-chain fatty acids, Demyelination, Remyelination, Oligodendrocyte, Multiple sclerosis

## Abstract

**Background:**

The association of gut microbiota and diseases of the central nervous system (CNS), including multiple sclerosis (MS), has attracted much attention. Although a previous analysis of MS gut microbiota revealed a reduction in species producing short-chain fatty acids (SCFAs), the influence of these metabolites on demyelination and remyelination, the critical factors of MS pathogenesis, remains unclear.

**Methods:**

To investigate the relationship between demyelination and gut microbiota, we administered a mixture of non-absorbing antibiotics or SCFAs to mice with cuprizone-induced demyelination and evaluated demyelination and the accumulation of microglia. To analyze the direct effect of SCFAs on demyelination or remyelination, we induced demyelination in an organotypic cerebellar slice culture using lysolecithin and analyzed the demyelination and maturation of oligodendrocyte precursor cells with or without SCFA treatment.

**Results:**

The oral administration of antibiotics significantly enhanced cuprizone-induced demyelination. The oral administration of butyrate significantly ameliorated demyelination, even though the accumulation of microglia into demyelinated lesions was not affected. Furthermore, we showed that butyrate treatment significantly suppressed lysolecithin-induced demyelination and enhanced remyelination in an organotypic slice culture in the presence or absence of microglia, suggesting that butyrate may affect oligodendrocytes directly. Butyrate treatment facilitated the differentiation of immature oligodendrocytes.

**Conclusions:**

We revealed that treatment with butyrate suppressed demyelination and enhanced remyelination in an organotypic slice culture in association with facilitating oligodendrocyte differentiation. Our findings shed light on a novel mechanism of interaction between the metabolites of gut microbiota and the CNS and may provide a strategy to control demyelination and remyelination in MS.

## Background

Multiple sclerosis (MS) is a chronic demyelinating inflammatory disease of the central nervous system (CNS). During the early stage of MS, most patients exhibit a relapsing-remitting disease course (relapsing-remitting MS (RRMS)). However, at the later stages of the disease, some patients enter a secondary-progressive phase characterized by the accumulation of irreversible neurological disabilities (secondary-progressive MS (SPMS)) [1]. Although the etiology and pathogenesis of MS remain to be elucidated, epidemiological studies have revealed that both genetic and environmental factors are involved in its development [2]. It has long been postulated that MS is an autoimmune disease mediated by T cells reactive to myelin autoantigens, such as myelin basic protein [3]. Recent genome-wide association studies revealed a major role for cellular autoimmunity in MS because many genes associated with the differentiation, activation, and proliferation of CD4^+^ helper T cells have been linked to MS susceptibility [4–7]. The efficacy of therapy to block the entry of T cells into the CNS of MS patients also supports the importance of T cells in the pathogenesis of relapsing-remitting RRMS [8]. However, the therapeutic effect of these drugs is limited in SPMS and the insufficiency of remyelination and subsequent degeneration of neurons persist in the brain of SPMS patients [9]. Therefore, unraveling the mechanism of impaired remyelination might contribute to the development of a novel therapy for progressive MS.

Recently, the association of gut microbiota and various CNS diseases including neurodegenerative diseases, psychiatric diseases, and neuroinflammatory diseases such as MS have attracted attention [10–12]. We and other groups have reported the presence of dysbiosis in the gut microbiota of MS patients [13–20]. We found a reduction in bacteria belonging to *Clostridia* clusters IV and XIVa, which produce short-chain fatty acids (SCFAs) in MS patients [13]. Jangi et al. also reported that a SCFA-producing genus, *Butyricimonas*, was reduced in treated and untreated MS patients, suggesting the reduction of *Butyricimonas* may not be a secondary phenomenon of MS [14]. SCFAs are defined as groups of fatty acids with fewer than six carbons, especially acetate, propionate, and butyrate [21]. Indigestible dietary fiber usually metabolized by microbiota in the cecum and colon and SCFAs are major metabolites produced from microbial fermentative activity. SCFAs were reported to have many important roles in the maintenance of gut health, the control of energy metabolism, and regulation of the immune system [22, 23]. Several reports have demonstrated that SCFAs regulate gut immunity by inducing regulatory T cells (Treg cells) through the inhibition of histone deacetylase (HDAC) [24–26]. Butyrate was also reported to act as a ligand of the G-protein-coupled receptor, GPR109a, expressed on dendritic cells (DC), and to induce the production of retinoic acid and IL-10, which lead to the expansion of Treg cells [27]. We and other groups recently revealed that the oral administration of SCFAs ameliorated the disease severity of experimental autoimmune encephalomyelitis (EAE), an animal model of MS [28, 29]. Various effects of SCFAs on the CNS have also been shown. The permeability of the blood-brain barrier (BBB) was increased in germ-free mice and restored by the colonization of SCFA-producing bacteria [30]. In germ-free mice, microglia in the brain had an immature phenotype and branched cell shape and oral treatment with SCFAs restored microglial immaturity and malformation [31]. However, the relationship between gut microbiota or SCFAs and demyelination or remyelination remains unclear. The prevalence of MS but not neuromyelitis optica (NMO), an autoimmune astrocytopathy induced by anti-aquaporin-4 (AQP4) antibody, has increased in Japan over the last 30 years [32]. Therefore, we hypothesized that dysbiosis caused by dietary change is involved in demyelination and/or remyelination.

In this study, we revealed that the oral administration of non-absorbing antibiotics significantly exacerbated cuprizone-induced demyelination and the oral administration of butyrate significantly ameliorated cuprizone-induced demyelination. We further demonstrated that butyrate treatment significantly suppressed lysolecithin (LPC)-induced demyelination and increased remyelination in association with the enhanced differentiation of immature oligodendrocytes. In addition, the depletion of microglia did not affect the butyrate-mediated suppression of demyelination and enhancement of remyelination, suggesting that butyrate directly affected the maturation of oligodendrocytes. Our findings report a new mechanism related to how the gut environment affects homeostasis in the CNS.

## Material and methods

### Cuprizone, antibiotic, and SCFA treatment of mice

Six-week-old C57BL/6 J (B6) male mice purchased from The Jackson Laboratory (Bar Harbor, ME) were used for the following series of experiments. This study was approved by the Animal Experimental Committee of Juntendo University Graduate School of Medicine. Mice were maintained in specific pathogen-free conditions in accordance with institutional guidelines. All mice were sacrificed under isoflurane anesthesia. For cuprizone-induced demyelination, B6 male mice were fed a chow diet including 0.2% cuprizone (bis-cyclohexanone oxaldihydrazone; Sigma, St. Louis, MO) for 3 weeks allowing the acute phase of demyelination to occur. Control mice were fed standard chow. For antibiotic treatment, we administered non-absorbing antibiotics to mice as previously reported [33]. Briefly, mice were given ad libitum access to drinking water supplemented with kanamycin (1 mg/ml), colistin (0.08 mg/ml), and vancomycin (0.1 mg/ml) for 1 week before the cuprizone diet began and throughout the whole experiment. For treatment with SCFAs, mice were given acetate, propionate, and butyrate (Wako Pure Chemical Industries, Osaka, Japan) added to the drinking water at a concentration of 200 mM for 1 week before the cuprizone diet began and throughout the whole experiment. Control mice were fed normal drinking water.

### Brain tissue processing

Mice were transcardially perfused with 4% paraformaldehyde (PFA) under isoflurane anesthesia. Brains were surgically removed and fixed in 4% PFA overnight, then dehydrated proportionally using 5%, 10%, and 20% sucrose in phosphate-buffered saline solution, and finally embedded with O.C.T. compound (Sakura Finetek, Tokyo, Japan).

### Black-Gold II and immunofluorescence staining of brain tissues

For myelin staining, frozen parasagittal sections of brain tissue were cut at the corpus callosum at a thickness of 20 μm. Two sections were stained per mouse using the Black-Gold II myelin staining kit (Millipore, Billerica, MA) according to the manufacturer’s protocol. Images of brain sections were captured by a BZ-X700 fluorescence microscope (Keyence, Tokyo, Japan) and the percentage of myelinated area within the corpus callosum was quantified using ImageJ software (National Institutes of Health, Bethesda, MA).

For immunofluorescence staining, brain sections were cut at 14 μm thickness. After blocking with 10% normal donkey serum (Jackson ImmunoResearch, West Grove, PA) for 1 h at room temperature, brain sections were incubated with primary antibodies against Iba-1 (Wako, 1:500) and CD68 (clone FA-11, Bio-Rad, Hercules, CA; 1:200) overnight. TRITC donkey anti-rabbit IgG (Jackson ImmunoResearch, 1:200) and Alexa Fluor 488 donkey anti-rat IgG (Jackson ImmunoResearch, 1:400) were used as secondary antibodies. Nuclei were stained with 4,6-diamidino-2-phenylindole (DAPI, Vector Laboratories, Burlingame, CA). All images were captured with a BZ-X700 fluorescence microscope (Keyence).

### Organotypic slice culture

Brain slices were prepared from postnatal day 9–10 B6 mice. After decapitation, the cerebellum was removed and sagittally sliced to a 300 mm thickness with a McIlwain tissue chopper (The Mickle Laboratory Engineering, UK). The cerebellum slices were transferred onto porous translucent membrane inserts (Millicell-CM: PICM03050, Millipore, Billerica, MA) in six-well culture plates at four to five slices per insert. Slices were cultured for 6 days with slice culture medium consisting of 49% Opti-MEM (Thermo Fisher Scientific, Waltham, MA), 25% Hank’s balanced salt solution (Thermo Fisher Scientific), 25% heat-inactivated horse serum, 5 mg/ml D-glucose (Wako Chemicals, Tokyo, Japan), and 1% penicillin/streptomycin (Thermo Fisher Scientific). For demyelination studies, demyelination was induced by 24 h incubation in 0.5 mg/ml LPC with or without butyrate. After demyelination, slices were washed and incubated with normal slice culture medium for 72 h and fixed with 4% PFA for 45 min. For remyelination studies, demyelination was induced by 24 h incubation in 0.5 mg/ml LPC. Slices were washed and incubated in normal slice culture medium with or without butyrate for 7 days. Slices were fixed with 4% PFA for 45 min. After fixation, slices were blocked and permeabilized with 2% bovine serum albumin (Iwai Chemicals, Tokyo, Japan) and 0.5% Triton X-100 (Wako). Slices were incubated with primary antibodies against myelin basic protein (MBP; BioLegend, San Diego, CA), neurofilament-200 (NF200; Sigma-Aldrich, St. Louis, MO), platelet-derived growth factor receptor alpha chain (PDGFR-α; BD Bioscience, Franklin Lakes, NJ), Olig2 (Abcam, Cambridge, UK), and CC-1 (Abcam) overnight at 4 °C. Secondary antibodies (FITC donkey anti-mouse IgG, Alexa 647 donkey anti-rat IgG, and rhodamine goat anti-rabbit IgG; Jackson ImmunoResearch) were incubated for 1 h at room temperature. Slices were mounted with Vectashield HardSet Mounting Medium (Vector Laboratories, Burlingame, CA). Images were acquired using an FV1000-D microscope (Olympus, Tokyo, Japan). For each group, 12 fields were randomly selected and photographed by fluorescence microscopy using a digital camera. For quantification, the myelination index (MI) was calculated using the following formula: MI = MBP-NF200 colocalization area/NF200-stained area. NF200-stained and MBP-NF200 colocalization areas were measured using ImageJ software.

### Real-time quantitative PCR

Total RNA was purified using RNeasy mini kit (QIAGEN, Hilden, Germany). cDNA was synthesized from 500 ng total RNA using ReverTra Ace qPCR RT Master Mix (TOYOBO, Osaka, Japan). Real-time quantitative PCR (RT-qPCR) was performed in 7500 Fast Real-Time PCR System (Applied Biosystems, Foster City, CA) with Fast SYBR Green Master Mix (Thermo Fisher Scientific). mRNA levels were normalized to endogenous glyceraldehyde-3-phosphate dehydrogenase (GAPDH) in each sample. The specific primers used in this study are as follows: Gapdh sense, 5′-GGTTGTCTCCTGCGACTTCA-3′; Gapdh antisense, 5′-GCCGTATTCATTGTCATACCAGG-3′; Arg1 sense, 5′-GGAACCCAGAGAGAGCATGAG-3′; Arg1 antisense 5′-CTCGAGGCTGTCCTTTTGAGA-3′; Nos2 sense, 5′-AGGTCTTTGACGCTCGGAAC-3′; and Nos2 antisense 5′-GTCATGTTTGCCGTCACTCC-3′.

### Statistical analysis

Statistical analysis was performed using GraphPad Prism v8.1.2. Differences between data groups were analyzed by Mann-Whitney test or Kruskal-Wallis test followed by Dunn’s multiple comparisons test. *p* values less than 0.05 were considered significant.

## Results

### Butyrate suppresses cuprizone-induced demyelination

**Fig. 1:**
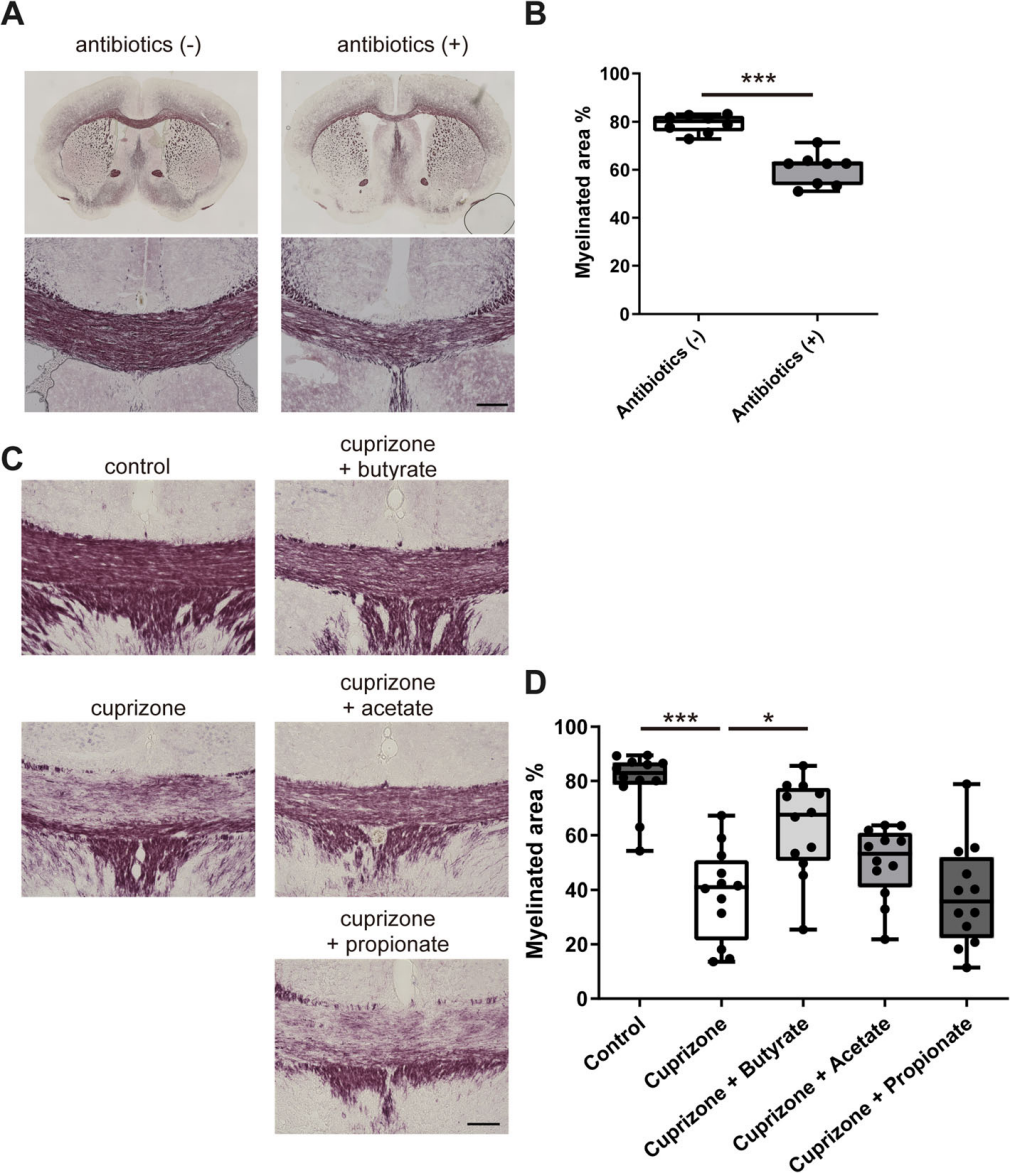
Oral treatment with antibiotics exacerbates cuprizone-induced demyelination and oral treatment of SCFAs suppresses demyelination in the corpus callosum. **a** Black-Gold staining of brains from cuprizone and antibiotic-treated mice. Scale bar, 100 μm. **b** Myelinated area of the corpus callosum (*n* = 8 mice per group, pooled from two independent experiments). **c** Black-Gold staining of brains from cuprizone and SCFA-treated mice. Scale bar, 100 μm. **d** Myelinated area of the corpus callosum (*n* = 12 mice per group, pooled from four independent experiments). The box plot indicates the first and third quartiles and the middle line indicates the median. Whiskers indicate the minimum and maximum. *p < 0.05, ***p < 0.001

To investigate the effect of intestinal dysbiosis on the function of oligodendrocytes, we induced a cuprizone-induced demyelinating mouse model, which lacks infiltrating peripheral T and B lymphocytes, to eliminate the potential effect of immunological mechanisms on demyelination. We started the oral administration of a non-absorbing antibiotic mixture (kanamycin, colistin, and vancomycin in drinking water) at 1 week before treatment with cuprizone. After 3 weeks of cuprizone treatment, we evaluated the demyelination of the corpus callosum. The myelinated areas of the corpus callosum were significantly reduced in the brains from antibiotic-treated mice compared with those from non-treated mice (Fig. 1a, b). This result indicated that intestinal dysbiosis induced by the antibiotics affected the cuprizone-induced demyelination. Next, we examined the effect of major metabolites of microbiota, SCFAs, on the cuprizone-induced demyelination. We administered SCFAs (acetate, propionate, or butyrate; 200 mM each in drinking water) to mice at 1 week before treatment with cuprizone and evaluated the demyelination of the corpus callosum after 3 weeks of cuprizone treatment. The myelinated areas of the corpus callosum were significantly reduced in the brains from cuprizone-treated mice compared with those from control mice (Fig. 1c, d). The myelinated areas of the corpus callosum in brains from butyrate-treated mice were significantly ameliorated compared with those in mice treated with cuprizone alone. Oral treatment with acetate showed mild improvement, but the difference did not reach statistical significance. Oral treatment with propionate did not ameliorate demyelination. These data suggested that gut microbiota may affect cuprizone-induced demyelination via their metabolites.

### Microglia are not affected by SCFA treatment in cuprizone-induced demyelination

**Fig. 2:**
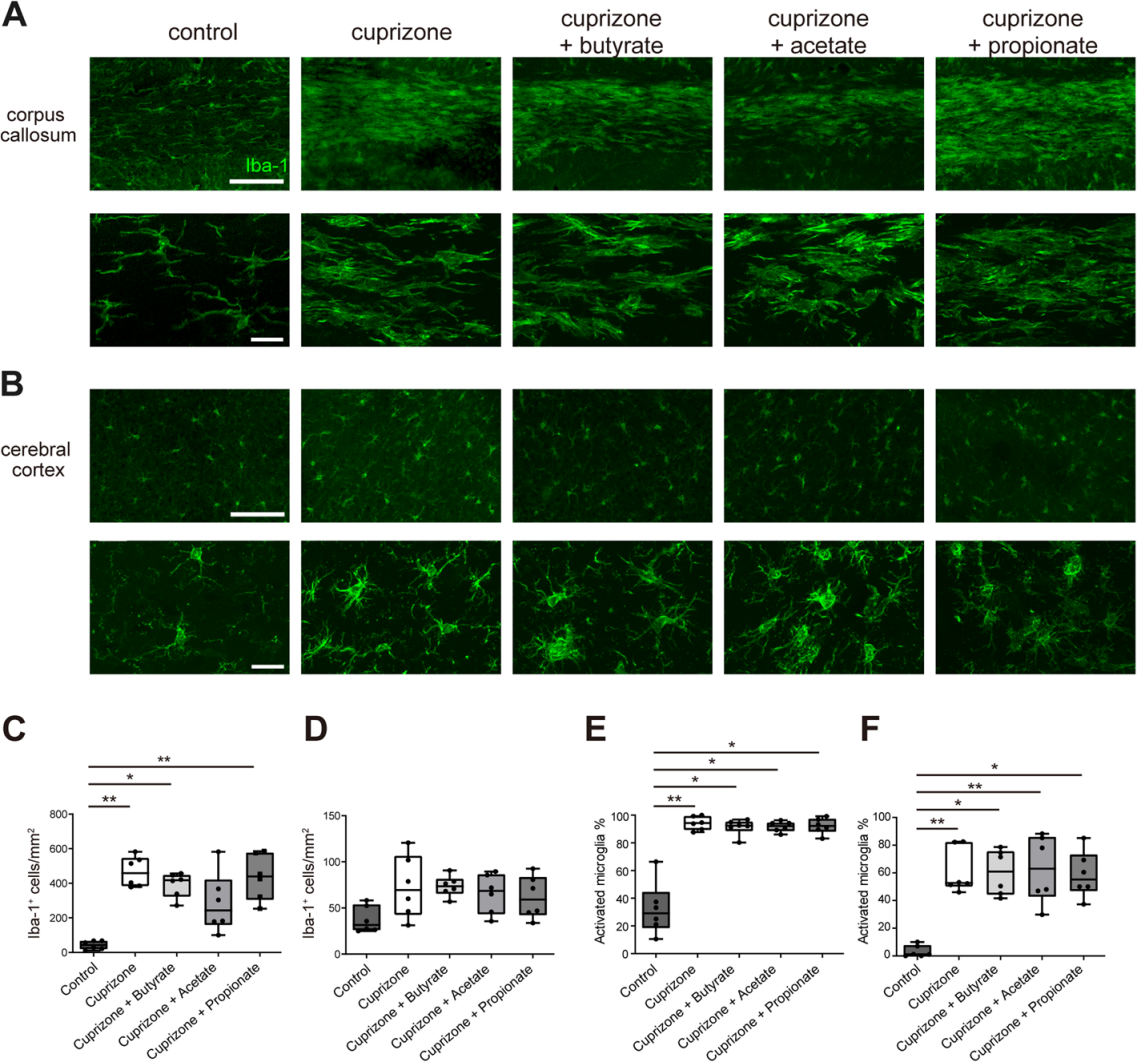
Microglia are not affected by oral treatment with SCFAs. **a** Iba-1 staining of the corpus callosum. Scale bar, 100 μm. The lower row indicates a magnified figure of microglia. Scale bar, 20 μm. **b** Iba-1 staining of the corpus cerebral cortex. Scale bar, 100 μm. The lower row indicates a magnified figure of microglia. Scale bar, 20 μm. **c** The number of Iba-1^+^ cells in the corpus callosum (*n* = 6 mice per group, pooled from two independent experiments). **d** The number of Iba-1^+^ cells in the cerebral cortex (*n* = 6 mice per group, data are representative of two independent experiments). **e** Percentage of CD68^+^ activated microglia in the corpus callosum (*n* = 6 mice per group, pooled from two independent experiments). **f** Percentage of CD68^+^ activated microglia in the cerebral cortex (*n* = 6 mice per group, pooled from two independent experiments). The box plot indicates the first and third quartiles and the middle line indicates the median. Whiskers indicate the minimum and maximum. *p < 0.05, **p < 0.01

In the cuprizone-induced demyelination model, microglia affect demyelination through cytokine production or phagocytosis [34, 35]. Recently, the oral treatment of SCFAs was reported to restore the immature phenotype of microglia in germ-free mice [31]. To analyze the effect of SCFA treatment on microglia during cuprizone-induced demyelination, we evaluated microglial accumulation into the demyelinating corpus callosum and cerebral cortex using immunohistochemistry. In the corpus callosum, cuprizone-treated mice had significantly increased microglial numbers compared with control mice (Fig. 2a, c). In SCFA-treated mice, the microglial number in the corpus callosum tended to be decreased by oral treatment with acetate, but the difference did not reach statistical significance compared with mice treated with cuprizone alone (Fig. 2a, c). The microglial number in propionate- or butyrate-treated mice was not decreased compared with mice treated with cuprizone alone (Fig. 2a, c). We also measured the number of microglia in the cerebral cortex to evaluate microglial accumulation in a mild demyelinated area. The cuprizone-treated mice had a slightly increased number of microglia in the cerebral cortex compared with control mice. The microglial number in the cortex of SCFA-treated mice was similar to that of cuprizone-treated mice (Fig. 2b, d). Morphologically, microglia in cuprizone-treated mice appeared to have an ameboid shape in the corpus callosum and cortex, whereas microglia in control mice had a ramified shape. Microglia in SCFA-treated mice also displayed an ameboid shape similar to mice treated with cuprizone alone (Fig. 2a, b). We also analyzed the proportion of CD68^+^-activated microglia in the corpus callosum and cortex. The proportion of activated microglia in the corpus callosum and cerebral cortex was not affected by oral treatment with SCFAs (Fig. 2e, f). Our data suggest that oral treatment with SCFAs did not reduce the number or activation status of microglia in demyelinated lesions. Therefore, the amelioration of demyelination by butyrate treatment did not appear to be related to the modulation of microglia.

### Butyrate suppresses demyelination and enhances remyelination in an organotypic cerebellar slice culture

**Fig. 3:**
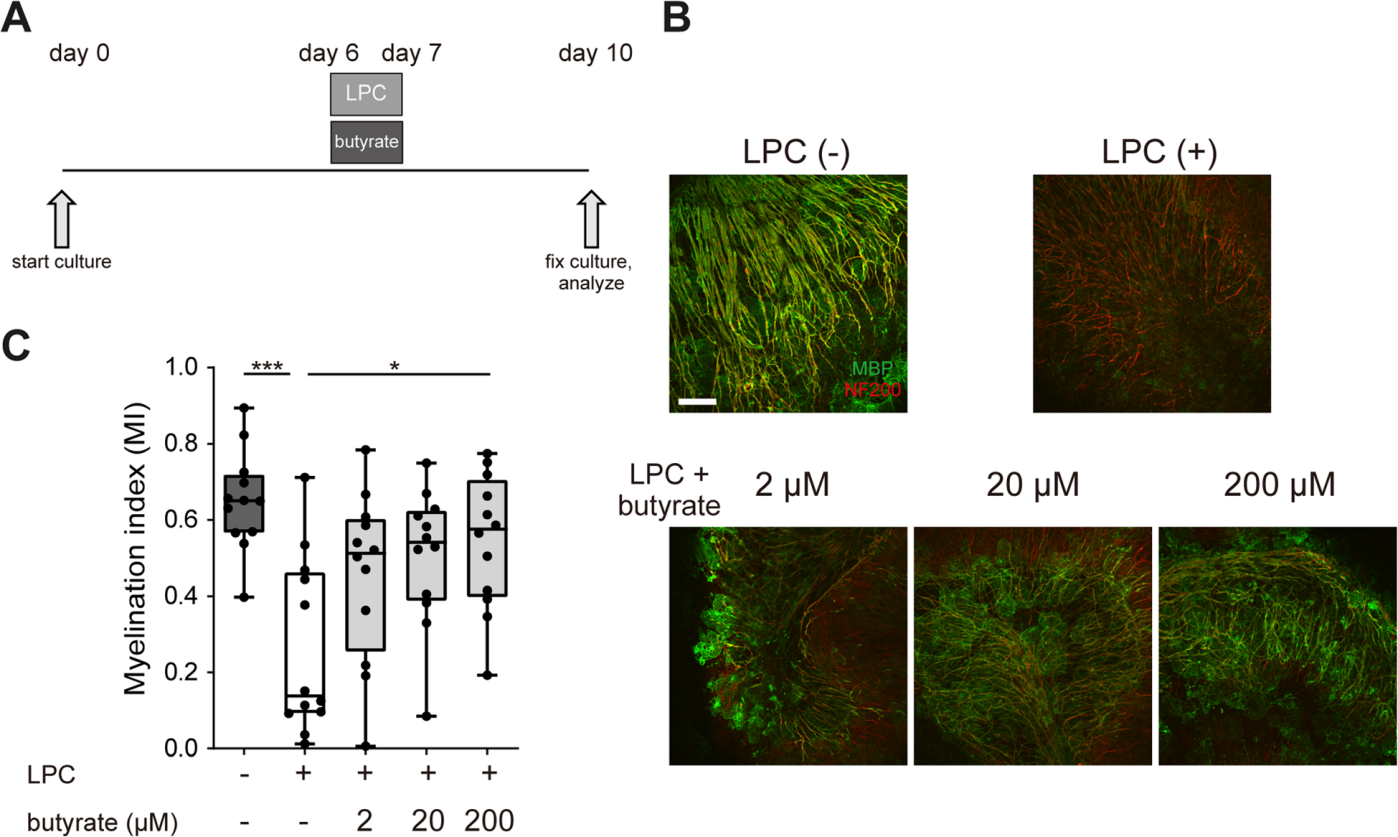
Butyrate treatment ameliorates LPC-induced demyelination in vitro*.* **a** Schematic showing the experimental protocol for demyelination analysis. **b** MBP and NF200 immunocytochemical staining of organotypic cerebellar slice cultures. Scale bar, 100 μm. **c** Myelination index (MI) of organotypic cerebellar slice cultures (*n* = 12 slices per group, pooled from four independent experiments). The box plot indicates the first and third quartiles and the middle line indicates the median. Whiskers indicate the minimum and maximum. *p < 0.05, ***p < 0.001

**Fig. 4:**
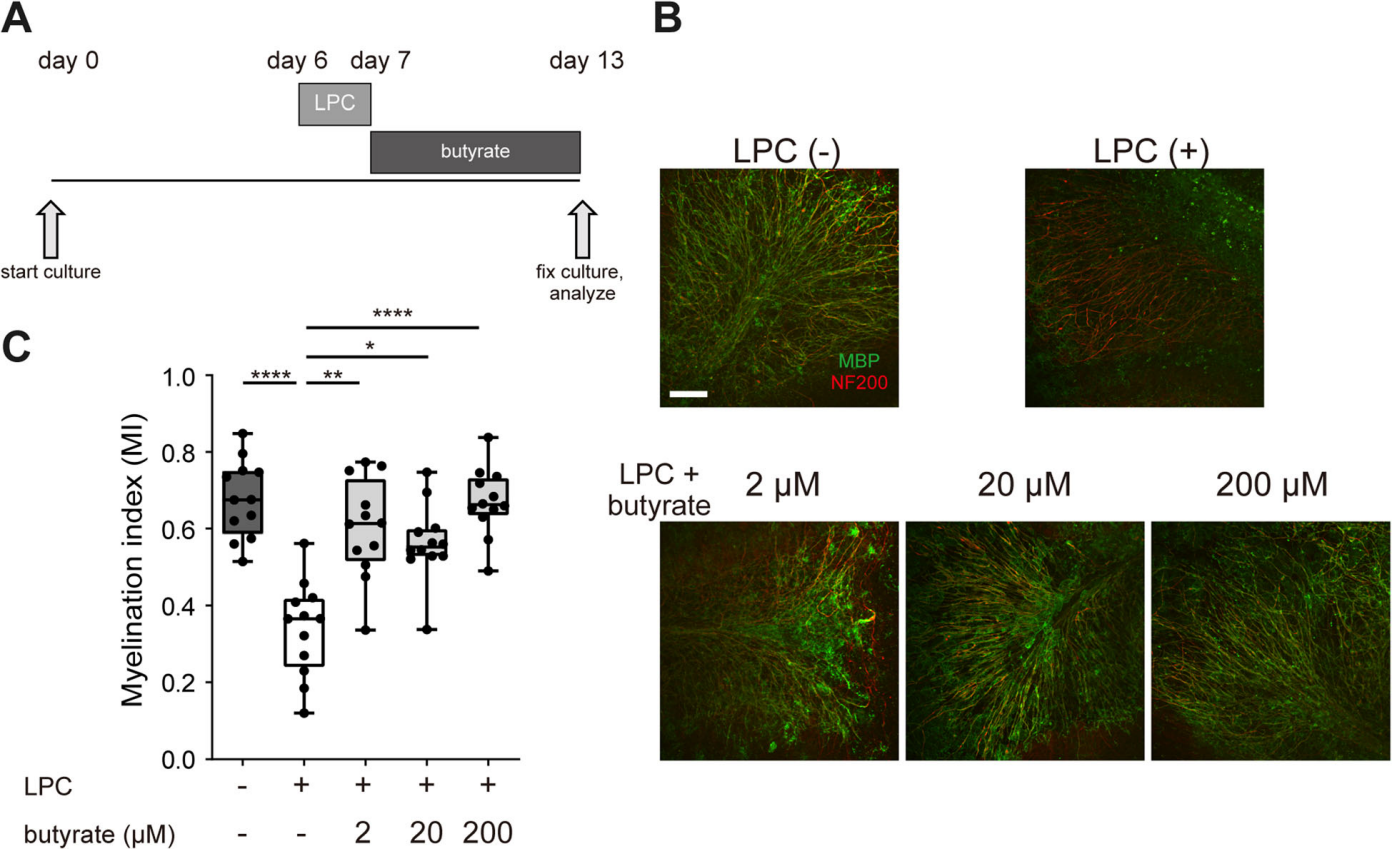
Butyrate treatment enhances remyelination from LPC-induced demyelination in vitro*.* **a** Schematic showing the experimental protocol for remyelination analysis. **b** MBP and NF200 immunocytochemical staining of organotypic cerebellar slice cultures. Scale bar, 100 μm. **c** Myelination index (MI) of organotypic cerebellar slice cultures (*n* = 3 slices per group, pooled from four independent experiments). The box plot indicates the first and third quartiles and the middle line indicates the median. Whiskers indicate the minimum and maximum. *p < 0.05, **p < 0.01, ****p < 0.0001

Recently, Treg cells were reported to facilitate remyelination in vivo [36]. In the cuprizone model, the possibility that butyrate enhances Treg cells infiltration cannot be excluded. To exclude the influence of Treg cells and assess the direct effect of butyrate on demyelination and remyelination, we used an organotypic cerebellar slice culture. We prepared the cerebellar slice culture from P9–10 mice and induced demyelination with LPC 6 days later. At the same time, we treated slice cultures with butyrate for 24 h. We then replaced the medium and cultured it for 72 h with normal medium. Cultured slices were fixed, and we evaluated the proportion of myelinated axons (Fig. 3a). Butyrate treatment significantly suppressed demyelination by a dose-dependent mechanism (Fig. 3b, c). To investigate the effect of butyrate on remyelination, we induced demyelination and then treated the demyelinated cerebellar slice culture with butyrate for 6 days. Cultured slices were fixed, and we evaluated the proportion of myelinated fibers present (Fig. 4a). Butyrate treatment significantly enhanced remyelination at all concentrations tested (Fig. 4b, c). These results indicated that butyrate directly suppressed demyelination and enhanced remyelination in vitro.

### Microglia depletion does not affect the butyrate-mediated suppression of demyelination and enhancement of remyelination

**Fig. 5:**
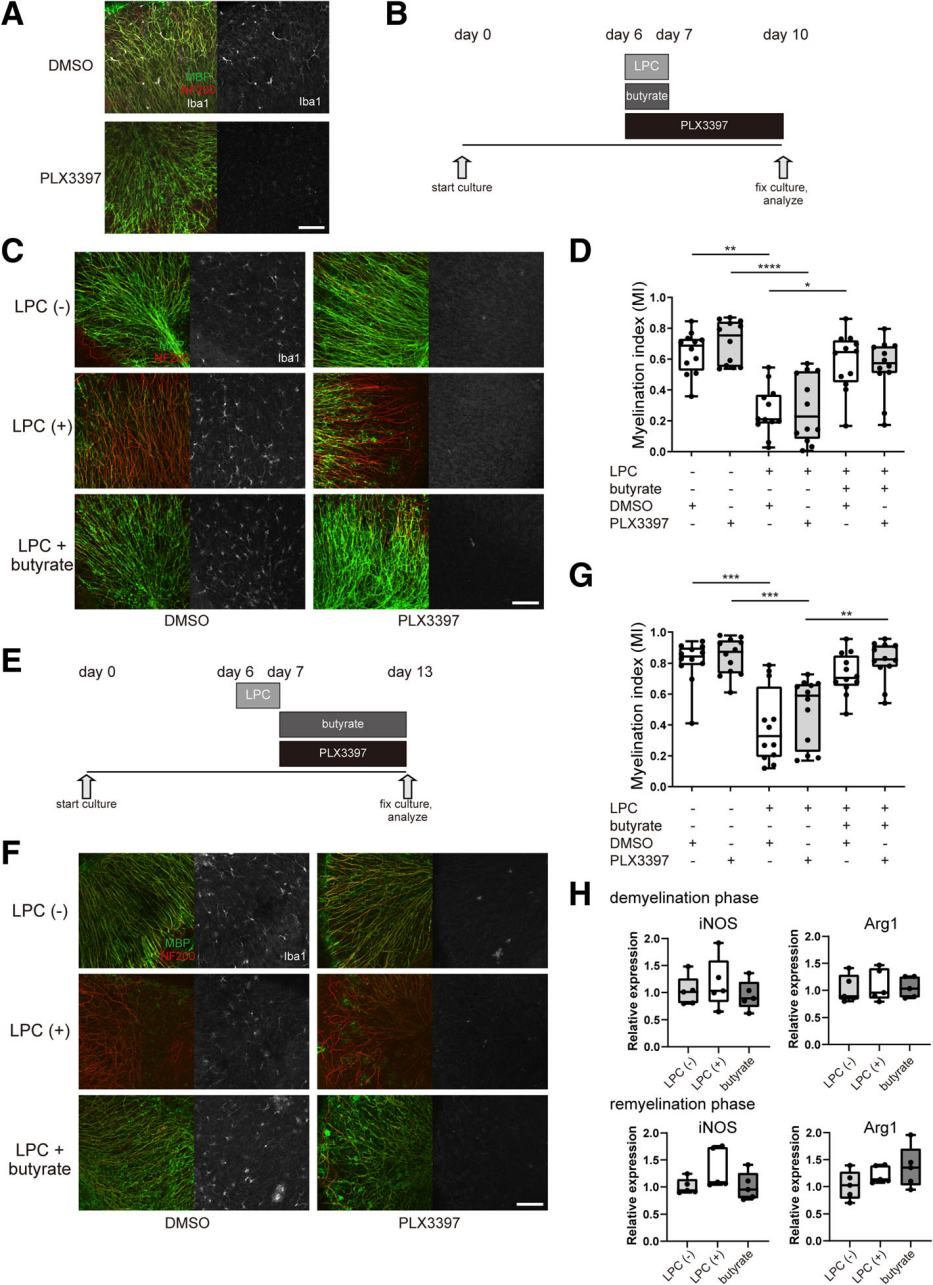
Depletion of microglia does not affect the butyrate-induced suppression of demyelination and enhancement of remyelination. **a** MBP, NF200, and Iba-1 immunocytochemical staining of PLX3397-treated or non-treated organotypic cerebellar slice cultures. Scale bar, 100 μm. **b** Schematic showing the experimental protocol of demyelination analysis. **c** Immunocytochemical staining of PLX3397-treated or non-treated organotypic cerebellar slice cultures with LPC and butyrate treatment. Scale bar, 100 μm. **d** Myelination index (MI) of organotypic cerebellar slice cultures (*n* = 12 slices per group, pooled from three independent experiments). **e** Schematic showing the experimental protocol of remyelination analysis. **f** Immunocytochemical staining of PLX3397-treated or non-treated organotypic cerebellar slice cultures with LPC and butyrate treatment. Scale bar, 100 μm. **g** MI of organotypic cerebellar slice cultures (*n* = 12 slices per group, pooled from three independent experiments). **h** relative expression of iNOS and Arg1 in organotypic slice cultures (*n* = 5 slice per group). The box plot indicates the first and third quartiles and the middle line indicates the median. Whiskers indicate the minimum and maximum. *p < 0.05, **p < 0.01, ***p < 0.001, ****p < 0.0001

To exclude the possibility that microglia affect the butyrate-mediated suppression of demyelination and enhancement of remyelination, we depleted microglia with PLX3397, an antagonist of colony-stimulating factor 1 receptor, through which signaling is necessary for microglial survival [37]. We confirmed that microglia in the cerebellar slice culture were almost completely depleted by the addition of PLX3397 (Fig. 5a). We treated cerebellar slice cultures with PLX3397 or vehicle during the demyelination phase (Fig. 5b) and observed that the depletion of microglia did not affect LPC-induced demyelination and suppression of demyelination mediated by butyrate (Fig. 5c, d). We also analyzed whether the depletion of microglia affected the butyrate-mediated enhancement of remyelination (Fig. 5e). No significant difference was observed between PLX3397 and vehicle treatment (Fig. 5f, g). To investigate whether butyrate treatment affects microglial phenotype, we analyzed the expression of M1 (inducible nitric oxide synthase: iNOS) and M2 (argnase-1 (Arg1)) markers in butyrate-treated or non-treated slice cultures using RT-qPCR. We found no difference in the expression of iNOS and Arg1 between butyrate-treated and non-treated slice cultures both in demyelination phase and remyelination phase (Fig. 5h). These data indicated that butyrate influenced demyelination and remyelination in the absence of microglia.

### Butyrate enhances the maturation of oligodendrocytes

**Fig. 6:**
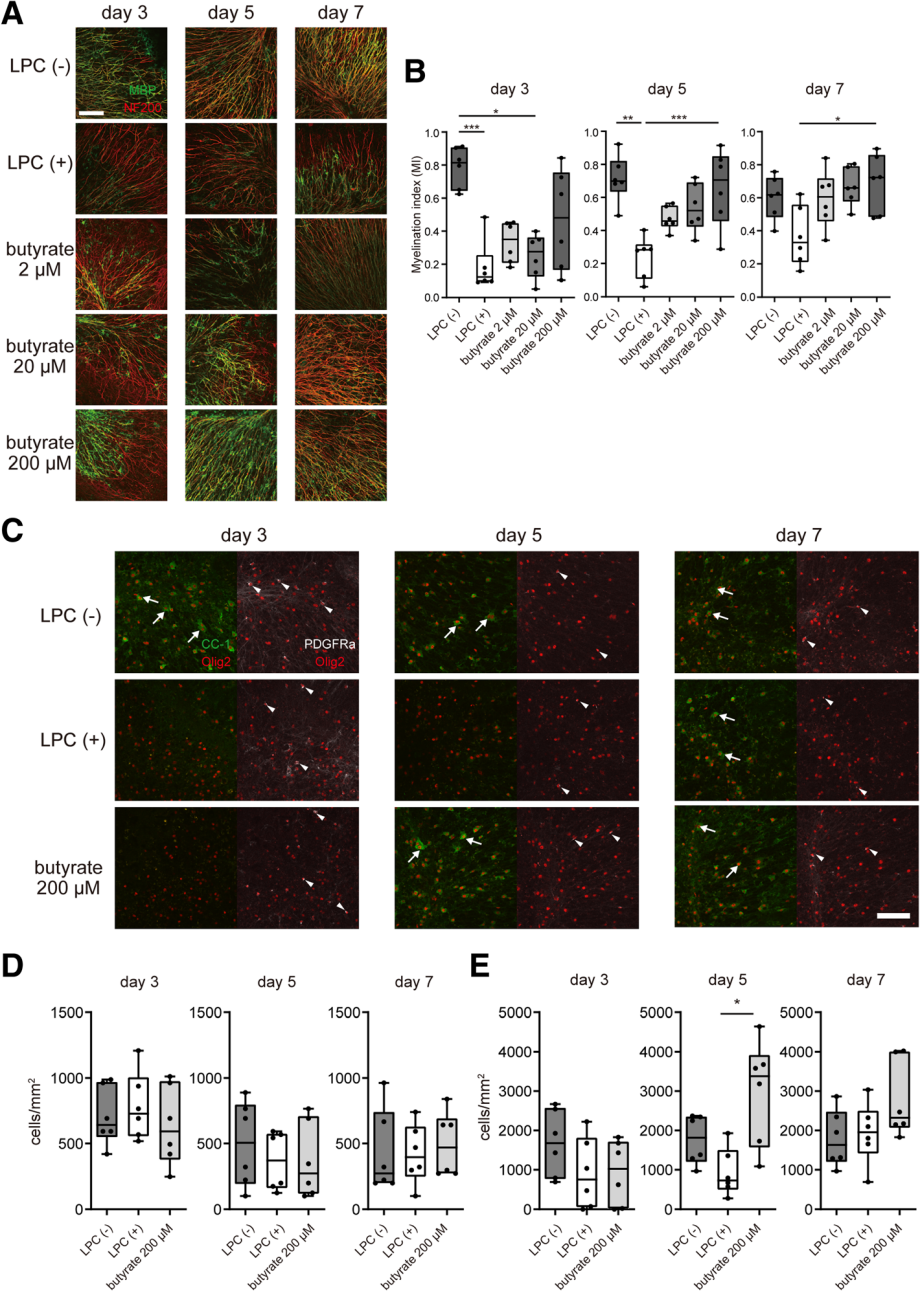
Butyrate treatment enhances the differentiation of oligodendrocyte. **a** MBP and NF200 immunocytochemical staining of organotypic cerebellar slice cultures. Slice cultures were treated with LPC for 24 h and then cultured with normal medium. At 3, 5, and 7 days after LPC treatment, cultures were fixed and analyzed. Scale bar, 100 μm. **b** Myelination index (MI) of organotypic cerebellar slice cultures (*n* = 6 slices per group, pooled from two independent experiments). **c** Olig2, CC-1, and PDGFRα immunocytochemical staining of organotypic cerebellar slice cultures. Slice cultures were treated with LPC for 24 h and then cultured with normal medium. At 3, 5, and 7 days after LPC treatment, cultures were fixed and analyzed. Arrows indicate Olig2^+^CC-1^+^ mature oligodendrocytes. Arrowheads indicate Olig2^+^PDGFRα^+^ OPC. Scale bar, 100 μm. **d** OPC number in the organotypic cerebellar slice culture (*n* = 6 slices per group, pooled from two independent experiments). **e** Mature oligodendrocyte number in the organotypic cerebellar slice culture (*n* = 6 slices per group, pooled from two independent experiments). The box plot indicates the first and third quartiles and the middle line indicates the median. Whiskers indicate the minimum and maximum. *p < 0.05, **p < 0.01, ***p < 0.001

To explore the mechanism of the butyrate-induced enhancement of remyelination, we examined oligodendrocyte maturation. We induced demyelination in an organotypic cerebellar slice culture with LPC and analyzed the effect of butyrate on remyelination sequentially (Fig. 6a, b). At 3 days after LPC-induced demyelination, butyrate treatment slightly enhanced the remyelination. At day 5, butyrate treatment significantly enhanced remyelination in a dose-dependent manner. Although the remyelination progressed gradually in butyrate- and non-treated cultures at day 7, butyrate-treated slices still showed a significant enhancement of remyelination compared with non-treated slices (Fig. 6a, b). Next, we investigated the influence of butyrate on oligodendrocyte maturation. We stained slice cultures with anti-Olig2 antibody, anti-CC-1 antibody, and anti-PDGFRα antibody and measured the numbers of Olig2-positive and CC-1-positive mature oligodendrocytes, and PDGFRα-positive and Olig2-positive oligodendrocyte precursor cells (OPC) (Fig. 6c–e). We revealed that butyrate treatment did not affect the number of OPC in the cerebellar slice cultures (Fig. 6c, d). However, the number of Olig2^+^CC-1^+^ mature oligodendrocytes in the LPC-treated culture was reduced at day 3. At day 5, the number of mature oligodendrocytes in the butyrate-treated culture was significantly increased compared with the non-treated culture. At day 7, the butyrate-treated culture showed an increased tendency to contain mature oligodendrocytes compared with the non-treated culture (Fig. 6c, e). These data indicated that butyrate treatment enhanced remyelination by accelerating oligodendrocyte maturation rather than by enhancing OPC proliferation.

### HDAC inhibitor suppresses LPC-induced demyelination and enhances remyelination

**Fig. 7:**
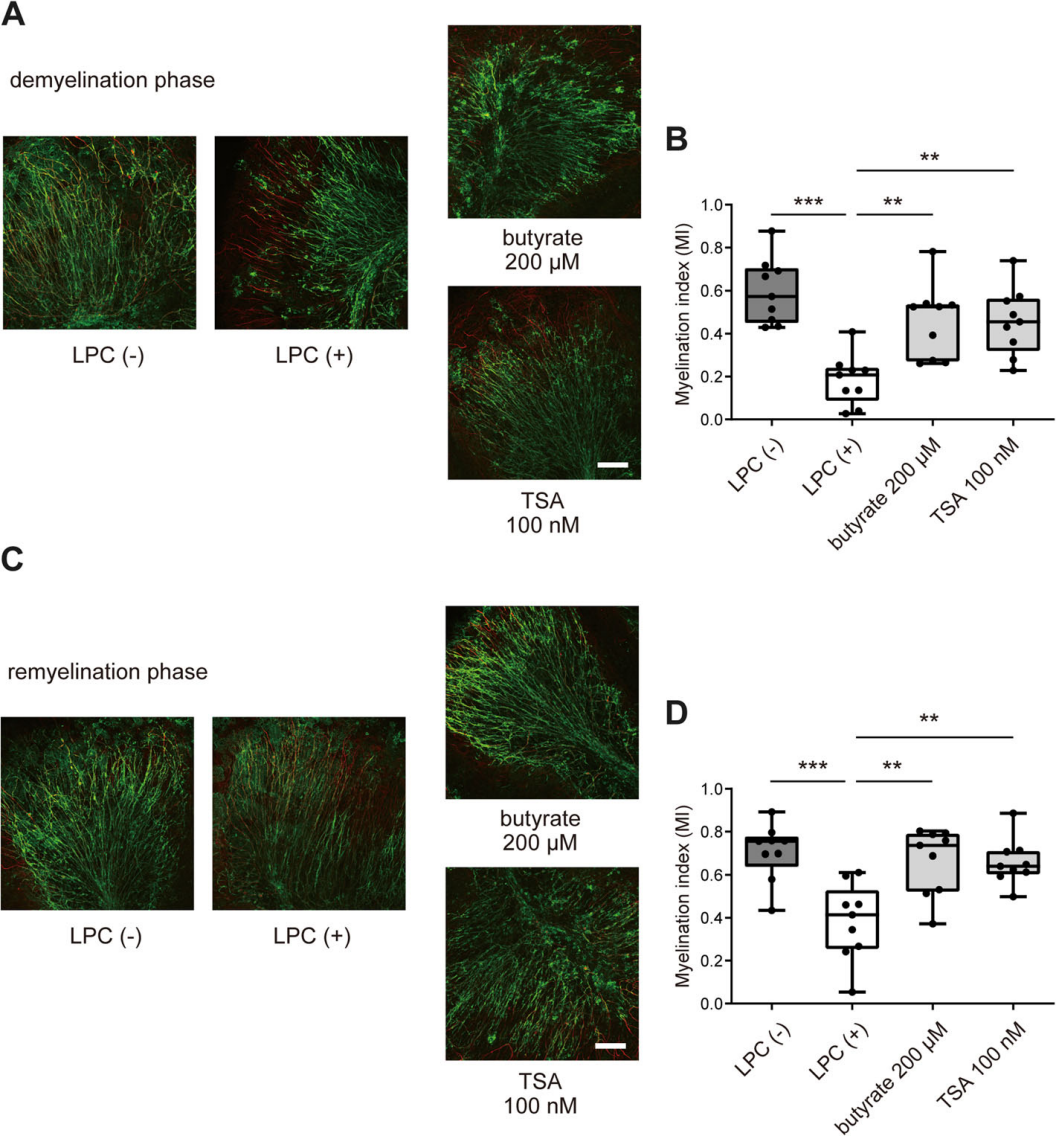
HDAC inhibitor suppresses LPC-induced demyelination and enhances remyelination. **a** MBP and NF200 immunocytochemical staining of organotypic cerebellar slice cultures with or without butyrate or TSA treatment in the remyelination phase. Scale bar, 100 μm. **b** Myelination index (MI) of organotypic cerebellar slice cultures (*n* = 9 slices per group, pooled from three independent experiments). **c** MBP and NF200 immunocytochemical staining of organotypic cerebellar slice cultures with or without butyrate or TSA treatment in the demyelination phase. Scale bar, 100 μm. **d** MI of organotypic cerebellar slice cultures (*n* = 9 slices per group, pooled from three independent experiments). The box plot indicates the first and third quartiles and the middle line indicates the median. Whiskers indicate the minimum and maximum. **p < 0.01, ***p < 0.001

Because butyrate is an HDAC inhibitor, we compared the effects of trichostatin A (TSA), an HDAC inhibitor, and butyrate on demyelination and remyelination. TSA treatment significantly suppressed LPC-induced demyelination similar to the butyrate treatment (Fig. 7a, b). In the remyelination phase, both butyrate and TSA treatment significantly improved remyelination from LPC-induced demyelination compared with the LPC-only treatment group (Fig. 7c, d). Our data revealed that the HDAC inhibitor suppressed demyelination and enhanced remyelination similar to butyrate and suggested that butyrate affects oligodendrocytes by acting as an HDAC inhibitor.

## Discussion

In this study, we demonstrated that gut dysbiosis induced by oral antibiotic treatment significantly exacerbated cuprizone-induced demyelination and that oral treatment with butyrate significantly ameliorated demyelination in vivo. The accumulation of microglia into demyelinated lesions was not affected by butyrate treatment, suggesting butyrate directly affected oligodendrocytes. Furthermore, we revealed that butyrate treatment suppressed LPC-induced demyelination and enhanced remyelination in association with the facilitation of oligodendrocyte maturation. These effects were independent of microglia and were assumed to be mediated by their activity as a HDAC inhibitor.

Recently, Tregs were reported to be involved in oligodendrocyte differentiation and remyelination through the production of CCN3 [36]. In the gut mucosa, Tregs play a crucial role in maintaining immune homeostasis and are induced by several microbial components or metabolites including SCFAs [24–26]. SCFAs, especially butyrate, enhance the expression of transcription factor forkhead box protein 3 (Foxp3), a key transcription factor of Tregs, by inducing the acetylation of histone H3 in the enhancer region of Foxp3 through the inhibition of HDAC. Oral treatment with SCFAs induces Tregs and therefore may affect cuprizone-induced demyelination. To exclude the involvement of Treg expansion in the gut, we used an organotypic slice culture and revealed that SCFA treatment inhibited demyelination and enhanced remyelination. These results suggested that SCFAs affect demyelination and remyelination independent of the expansion of Tregs in the gut.

Microglia, resident macrophages of the CNS, were reported to have contradictory roles in demyelination [34, 35]. In a cuprizone-induced demyelination model, microglia accumulated into demyelinating lesions and promoted an inflammatory response through the production of proinflammatory cytokines including tumor necrosis factor-α (TNF-α), interleukin-1β (IL-1β), and interferon-γ (IFN-γ) [38–40]. In contrast, microglia-derived soluble factors promoted the survival and maturation of oligodendrocytes and OPC in several in vitro studies [41–43]. Insulin-like growth factor 1 (IGF-1) was shown to play important roles in oligodendrocyte differentiation, survival, and myelination in various demyelinating models including cuprizone-induced demyelination [44]. Furthermore, IGF-1 mRNA expression in microglia was enhanced during cuprizone-induced demyelination [45]. In addition, the influence of gut microbiota on microglia was demonstrated [31]. Germ-free and antibiotic-treated mice showed increased numbers of microglia with an immature phenotype and abnormal morphology and these changes were restored by SCFA. In the present study, microglia accumulated into the demyelinated corpus callosum and the number of microglia in demyelinated lesions was not affected by treatment with SCFAs. Even though we analyzed microglial activation states using CD68 immunostaining, the number and proportion of CD68 expressing microglia in the corpus callosum and cerebral cortex were not affected by SCFAs. Furthermore, the depletion of microglia had no effect on the butyrate-mediated suppression of demyelination and enhancement of remyelination in our culture model. These results suggested that butyrate affects demyelination and remyelination in a microglial-independent manner.

In the current study, butyrate acted directly on oligodendrocytes to suppress demyelination and enhance remyelination. Regarding remyelination, we showed that butyrate facilitated the expansion of Olig2^+^CC-1^+^ mature oligodendrocyte without increasing OPC in organotypic slice cultures suggesting butyrate enhances the differentiation of oligodendrocytes after demyelination. It could be due to the protective effect of butyrate that prevents the death of mature oligodendrocyte and facilitate their survival. However, Crawford et al. revealed that pre-existing and surviving mature oligodendrocyte did not contribute to remyelination after LPC-induced demyelination in the spinal cord [46]. Considering the impairment of remyelination but not a decrease of OPC was reported in the brains of MS patients [47], dysbiosis of MS patients characterized by the deletion of SCFA-producing bacteria may influence the development and disease progression of MS.

Myelination in the prefrontal cortex and the expression of genes associated with myelination were reported to be affected by microbiota at developmental stages [48, 49]. However, there is very little evidence for a direct association between gut microbiota and oligodendrocytes, especially in the process of demyelination and remyelination. Bacterial metabolites, particularly butyrate, are known to inhibit HDAC. The association between oligodendrocytes and HDAC has been studied but their results remain controversial. Butyrate and other HDAC inhibitors suppressed ischemia-induced oligodendrocyte damage in rat brains through multiple mechanisms involving the suppression of microglia [50]. Wang et al. reported that HDAC inhibition prevented white matter injury through the modulation of microglial polarization in a traumatic brain injury model [51]. In this study, we revealed that both butyrate and TSA suppressed LPC-induced demyelination and enhanced remyelination. These results suggested that HDAC inhibitors might attenuate oligodendroglial damage and facilitate oligodendroglial maturation. However, the genetic deletion of HDAC1 and HDAC2 in mice induced insufficiency of oligodendrocyte development and myelination in the developmental stage [52]. HDAC inhibitors were reported to block the differentiation of human fetus-derived OPC [53], and suppress the survival of rat Schwann cells in vitro [54]. Dincman et al. reported the cytotoxicity of HDAC inhibitor, suberoylanilide hydroxamic acid, to OPC in vitro and in vivo [55]. This discrepancy may be explained by the difference of developmental stages. Although HDAC inhibition suppresses the survival and differentiation of OPC in fetus or early postnatal mouse, the maturation of OPC may be facilitated by the HDAC inhibitor after postnatal stage.

## Conclusions

In conclusion, we demonstrated that the oral administration of SCFAs ameliorated cuprizone-induced demyelination in vivo. Furthermore, treatment with SCFAs suppressed LPC-induced demyelination and enhanced remyelination in association with facilitating oligodendrocyte differentiation. Our results shed light on the association between gut microbiota metabolites, and the CNS, especially oligodendrocytes, and provides a new clue to control demyelination and remyelination in demyelinating diseases such as MS.

## Data Availability

The datasets used and/or analyzed for the current study are available from the corresponding author upon reasonable request.

## Abbreviations

AQP4: Aquaporin-4
Arg1: Arginase-1
B6: C57BL/6 J
BBB: Blood-brain barrier
CNS: Central nervous system
DAPI: 4,6-Diamidino-2-phenylindole
DC: Dendritic cells
EAE: Experimental autoimmune encephalomyelitis
GAPDH: Glyceraldehyde-3-phosphate dehydrogenase
HDAC: Histone deacetylase
IFN-γ: Interferon-γ
IGF-1: Insulin-like growth factor 1
IL-1β: Interleukin-1β
iNOS: Inducible nitric oxide synthase
LPC: Lysolecithin
MBP: Myelin basic protein
MI: Myelination index
MS: Multiple sclerosis
NF200: Neurofilament-200
NMO: Neuromyelitis optica
OPC: Oligodendrocyte progenitor cells
PDGFR-α: Platelet-derived growth factor receptor alpha chain
PFA: Paraformaldehyde
RRMS: Relapsing-remitting multiple sclerosis
RT-qPCR: Real-time quantitative PCR
SCFA: Short-chain fatty acid
SPMS: Secondary-progressive multiple sclerosis
TNF-α: Tumor necrosis factor-α
TSA: Trichostatin A
